# Source-To-Sink Transport of Sugar and Its Role in Male Reproductive Development

**DOI:** 10.3390/genes13081323

**Published:** 2022-07-25

**Authors:** Jingbin Li, Yu-Jin Kim, Dabing Zhang

**Affiliations:** 1 Joint International Research Laboratory of Metabolic and Developmental Sciences, State Key Laboratory of Hybrid Rice, School of Life Sciences and Biotechnology, Shanghai Jiao Tong University, Shanghai 200240, China; lijingbin528@gmail.com; 2 Department of Life Science and Environmental Biochemistry, Pusan National University, Miryang 50463, Korea; yjkim2020@pusan.ac.kr; 3 School of Agriculture, Food and Wine, University of Adelaide, Urrbrae, SA 5064, Australia

**Keywords:** sugar partitioning, phloem, sink, source, sugar signaling

## Abstract

Sucrose is produced in leaf mesophyll cells via photosynthesis and exported to non-photosynthetic sink tissues through the phloem. The molecular basis of source-to-sink long-distance transport in cereal crop plants is of importance due to its direct influence on grain yield—pollen grains, essential for male fertility, are filled with sugary starch, and rely on long-distance sugar transport from source leaves. Here, we overview sugar partitioning via phloem transport in rice, especially where relevant for male reproductive development. Phloem loading and unloading in source leaves and sink tissues uses a combination of the symplastic, apoplastic, and/or polymer trapping pathways. The symplastic and polymer trapping pathways are passive processes, correlated with source activity and sugar gradients. In contrast, apoplastic phloem loading/unloading involves active processes and several proteins, including SUcrose Transporters (SUTs), Sugars Will Eventually be Exported Transporters (SWEETs), Invertases (INVs), and MonoSaccharide Transporters (MSTs). Numerous transcription factors combine to create a complex network, such as DNA binding with One Finger 11 (DOF11), Carbon Starved Anther (CSA), and CSA2, which regulates sugar metabolism in normal male reproductive development and in response to changes in environmental signals, such as photoperiod.

## 1. Introduction

Rice (Oryza sativa), the monocot model plant, is a major crop, meeting the food demands of more than 50% of the global population [1,2]. Reproductive development, which connects the dominant diploid sporophytic and short haploid gametophytic stages, is a critical element in grain production [3]. The male reproductive organ, the *stamen*, consists of a filament and an anther containing multiple specialized tissues that generate mature male gametophytes, the *pollen grains*, via a series of developmental events such as meristem specification, cell differentiation, meiosis, mitosis, and starch accumulation [4,5]. Research on anther development is essential to increase our understanding of developmental biology and boost agricultural production reviewed by [4,5,6,7,8].

Sugars are the constituents of main anther, and play essential roles in cell structure formation, energy supply, and male fertility in response to environmental conditions [9]. In rice, the expression of *Cell Wall Invertase 3* (*OsCWIN3/OsINV4*) correlates with sucrose accumulation and pollen sterility depending on temperature [10], while two MYB domain proteins, Carbon Starved Anther (CSA) and CSA2, regulate sugar partitioning and male fertility in response to photoperiod [11,12,13,14]. The sugar transporter OsXa13/OsSWEET11 plays essential roles in pollen development and disease resistance against bacterial blight [15,16].

Sugar transport processes from source leaves to anther sinks are thus directly involved in male fertility. This review aims to provide an overview of sugar transport and its role in anther development, focusing on the molecular basis of sugar partitioning from source to sink, and recent findings on how sugar metabolism impacts anther development.

## 2. Strategies of Source-to-Sink Sugar Partitioning

Carbon is fixed from carbon dioxide into carbohydrate in chloroplasts of leaf tissues, primarily mesophyll cells, and accumulated in the cytosol of the same cells. The energy demands of sink tissues, such as roots, flowers, and seeds, drive the export of sugars from the leaf, mainly in the form of sucrose, via long-distance transport in plant vasculature, the *phloem* [17]. Over half of the photo-assimilates (50–80%) are exported from source leaves to maintain non-photosynthetic sink tissues [18]. Carbohydrate partitioning from source-to-sink tissues comprises three elements [19]: phloem loading of sugars from source tissues; transportation in the sieve element of the phloem; phloem unloading of sugars to sink tissues [20].

**Figure 1:**
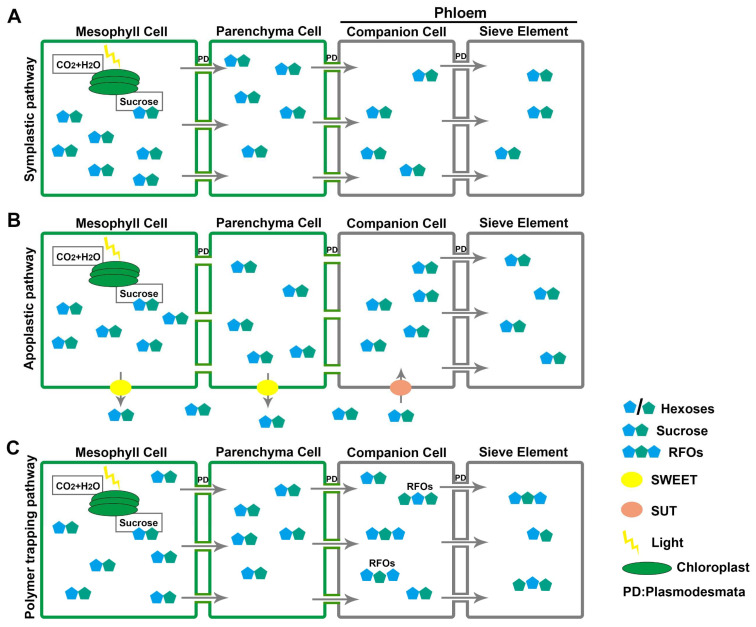
Three strategies for phloem loading. (**A**) Symplastic pathway: sucrose accumulates in mesophyll cells and is passively translocated to the phloem through plasmodesmata (PD) along the concentration gradient. (**B**) Apoplastic pathway: sucrose is exported to the apoplast by SWEETs and, after diffusion, imported into the phloem by SUTs. (**C**) Polymer trapping: sucrose is passively exported to phloem companion cells and synthesized into RFOs that can only move into sieve element cells due to their larger molecular mass.

Phloem is composed of several cell types, including parenchyma cells, sieve elements (SEs), and companion cells (CCs) [21,22]. Phloem loading is the first vital step in sugar’s long-distance transport—transferring the sugars from mesophyll cells to the SEs and CCs of the phloem [23,24,25]. Three different strategies are used for phloem loading by different plants according to the abundance of plasmodesmata, SUT activity, and the concentration gradient of photosynthates (Figure 1) [26].

The symplastic pathway is a passive loading process, driven by concentration gradients between mesophyll cells and phloem tissue (Figure 1A) [24,27], whereby the sucrose accumulated in mesophyll cells diffuses through plasmodesmata to reach phloem CCs [24,27]. Most tree species employ passive loading in the mesophyll cells, which meets the anatomical feature with high plasmodesmatal frequencies in the phloem of minor veins [26,28,29]. In most herbaceous plants, the apoplastic pathway is the main strategy for phloem loading [22]. Sucrose from mesophyll cells is actively exported to the apoplast by SWEET proteins (consuming energy), diffuses within the apoplast, and is actively loaded to phloem CCs via SUTs against a concentration gradient (Figure 1B) [22,28]. Polymer trapping, the third phloem-loading strategy, is an energy-consuming symplastic process adopted by a small number of specific plants (Figure 1C) [29]. For example, in *Arabidopsis* (*Arabidopsis thaliana*), cucumber (*Cucumis sativus*), and bugleweed (*Ajuga reptans*), sucrose diffuses into phloem CCs along the concentration gradient and is synthesized into raffinose-family oligosaccharides (RFOs); thus, larger sugars are retained in SE/CC complex due to the size of plasmodesmata [30,31]. In *Verbascum phoenicum*, RNA interference experiments demonstrate that the reduced expression of two *GAS* genes that drive RFO synthesis inhibits phloem transport and results in growth retardation [25].

## 3. Proteins Involved in Sugar Partitioning

### 3.1. Sucrose Transporters (SUTs)

Sucrose transporters (SUTs) act as symporters to import the sucrose from the apoplasm into phloem CCs against the concentration gradient, driven by the motive force generated by H^+^-ATPases (Figure 1B) [28,32]. The 12 transmembrane domains of the SUT protein forms a pore to transport sucrose across the plasma membrane [33].

The first sucrose transporter (*SoSUT*) was found in spinach (*Spinacea oleracea*) by an elegant yeast complementation strategy [34]. Nine and five SUTs have been found in *Arabidopsis* and rice, respectively [35,36]. Based on sequence, sub-cellular location, and activity, SUTs have been classified into three types: type I (specific to eudicots, plasma membrane–localized); type II (present in all plants, plasma membrane–localized); and type III (present in all plants, vacuolar membrane–localized) [37]. In rice, OsSUT1, OsSUT3, OsSUT4, and OsSUT5 are type II SUTs, and OsSUT2 is a type III tonoplast SUT (Table 1) [37].

### 3.2. Sugars Will Eventually Be Exported Transporters (SWEETs)

*SWEET*s are a group of evolutionally conserved genes expressed in eukaryotes, prokaryotes, and archaea [52,53]. These genes, encoding MtN3/saliva domain proteins, were initially found to encode glucose transporters [54], and have since been found to be capable of transporting a variety of mono- and di-saccharides [42,55,56,57]. According to their protein structures, SWEET proteins encode either one or two MtN3/saliva domains [58].

Rice encodes 21 SWEET proteins that are involved in multiple biological processes (Table 1) [58]. OsSWEET11, containing two MtN3/saliva domains, acts as a glucose uniporter in panicles and anthers [15]. Its knockdown mutant reveals defects in microspore development, suggesting a function in male development [15]. *OsSWEET11* is also upregulated in response to bacterial infection by *Xanthomonas oryzae pv. oryzae* [15]. *OsSWEET14* has a similar disease response, and its knockout mutant showed growth retardation, reduced plant size, and insensitivity to bacterial infection [16]. An overexpressing *OsSWEET5* line shows significant changes in leaf sugar levels, which indicates the function of *OsSWEET5* in sugar metabolism and transport [42]. This transgenic line also showed low expression of genes involved in auxin signal transduction, suggesting a function for SWEETs in regulating the crosstalk between auxin and sugar in rice [42]. In addition, five SWEET genes, including *OsSWEET1a*, *OsSWEET2a*, *OsSWEET4*, *OsSWEET11*, *OsSWEET15*, are highly expressed in panicles, indicating a putative function in sugar transport during rice reproductive development [59].

### 3.3. Invertases (INVs)

Invertases (INVs) encode proteins that hydrolyze sucrose into glucose and fructose [60], classified according to sub-cellular location into vacuolar (VIN), cell wall (CWIN), or cytoplasmic (CIN) invertases (Table 1) [61,62]. CINs prefer a neutral pH of 7.0–7.8 in the cytosol, while VINs and CWINs have an optimal pH of 4.5–5.5 [61]. Rice has 19 invertase genes, including nine *CWIN*s, two *VIN*s, and eight *VIN*s (Table 1) [63]. CWIN proteins bind to the cell wall and play essential roles in sugar transmembrane transport during phloem unloading [61].

### 3.4. Monosaccharide Transporters (MSTs)

Monosaccharide transporters (MSTs) are membrane proteins involved in the transmembrane transport of hexoses, hydrolyzed from sucrose by INVs, in sink tissues in the apoplastic pathway (Figure 1B) [64]. An *Arabidopsis* phylogeny of 53 MST proteins suggests seven subfamilies—AZT, XTPH, ERD, pGlcT, PLT, INT, and STP (Table 1)—many of whose expression patterns or function have not yet been characterized [65]. Among the seven subfamilies of MST proteins, AZT and XTPH proteins localize on the tonoplast and play essential roles in sugar transport to the tonoplast [66,67,68]. AtERD6, a member of ERD proteins, was proved to be involved in the transport of monosaccharides, whose expression was induced by abiotic stress [69]. pGlcT proteins are transporters of glucose, and PLT proteins are symporters of polyols and monosaccharides [70,71]. AtINT4, the first identified member of the INT proteins, exhibits H^+^ symporter activities for myoinositol in yeast (*Saccharomyces cerevisiae*) and *Xenopus laevis* oocytes [72]. MST members of the STP sub-family are H+/hexose cotransporters locating on plasma membranes, which transport a series of hexoses, including glucose, fructose, galactose, xylose, mannose, pentose, and ribose [64].

A slightly larger family of 64 MSTs has been found in rice; these proteins split into the same seven clades upon phylogenetic analysis [64]. Several members of the STP subfamily were reported to have monosaccharide transport functions (Table 1). OsMST3 is required to accumulate monosaccharides, the substrate for cellulose synthesis, during cell wall synthesis [46]. *OsMST5*, highly expressed in panicles, is associated with pollen growth [47]. Moreover, *OsMST8* is directly regulated by CSA, suggesting a function for *OsMST8* during anther development [11].

## 4. Roles of Sugar Transporters in Phloem Loading and Unloading

**Figure 2:**
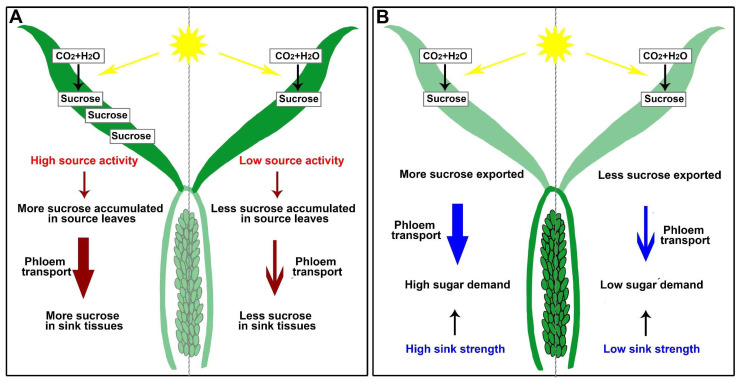
Schematic diagrams of sugar source-to-sink transport in rice. (**A**) High source activity in source leaves promotes phloem transport. (**B**) High sink strength results in high sugar demand, increasing the sugar transport.

Photosynthesis—“source activity”—and sink energy utilization—“sink strength”—combine to raise plant productivity [20,73]. Understanding the processes of phloem loading in source leaves and unloading in sink tissues can improve source activity and sink strength, leading to higher grain yields (Figure 2). After long-distance phloem transport from source tissues, sugar (mainly sucrose) is unloaded in sink organs; however, this process will lead to sucrose accumulation in sink tissues (reduced sink strength), resulting in reduced efficiency in sugar transport and source activity (Figure 2) [74].

In *Arabidopsis*, AtSUC2 is expressed in phloem CCs of minor leaf veins, which are supposed to be involved in the source-to-sink transition [75,76]. An *AtSUT2* T-DNA insertion mutant line exhibits decreased sucrose exports from leaves, resulting in sucrose accumulation in leaves, and delayed root growth and flowering [77]. OsSUT1, a type II SUT like AtSUC2, is highly expressed in leaves, stems, and grains; however, knockdown lines of OsSUT1 do not show sucrose accumulation in source leaves [38,39]. OsSUT3, another type II SUT, is preferentially expressed in pollen, suggesting a function in pollen development and maturity rather than phloem loading in source leaves [41]. OsSUT2, a type III SUT, is involved in sucrose transfer across the tonoplast from the vacuole lumen to the cytosol in rice [40].

SWEETs transport mono- or di-saccharides across membranes for phloem transport [78]. In *Arabidopsis*, AtSWEET11/12 localizes in the plasma membrane of vascular tissues and participates in phloem transport [55]. Maize ZmSWEET4c functions in hexose transport during seed development, and its mutation demonstrates a lack of hexose transport and defect in seed filling [79]. In rice, OsSWEET11 and OsSWEET14, two response factors to bacterial infection, also show essential roles in grain filling, whose mutants reveal defective in grain filling, resulting in increased starch accumulation in the pericarp [16,80,81]. OsSWEET15, another symporter highly expressed in rice caryopses, is necessary for sucrose efflux from caryopses to grains during seed filling [45]. OsSWEET5 encodes a galactose transporter, whose overexpression causes growth retardation and precocious senescence in rice seedlings [42]. These SWEET proteins showed important roles in grain filling, demonstrating their biological function of sucrose transfer from caryopses (source) to grains (sink).

Cell wall invertases (CWINs) play important roles in apoplasmic unloading, decreasing the concentration of sucrose in sink tissues to improve sink strength [61,82]. In rice, grain yield significantly decreased when the expression of *OsCWIN2* (*GIF1*) was suppressed [83], and a similar phenotype is observed in *ZmCWIN2* (*Incw2*) mutants in maize (*Zea mays*) [80]. *VfCWIN1* in *Vicia faba*, a dicot species, is also reported to impact seed size [81]. Moreover, *OsCWIN3* (*INV4*) has high expression in rice anthers, and affects male fertility in response to temperature variations [10].

During the apoplasmic unloading, sucrose is hydrolyzed into hexoses by CWINs, which results in monosaccharides accumulation in unloading tissues. Plasma membrane-localized MSTs are transporters of monosaccharides, which are responsible for hexoses partitioning to sink tissues [84]. In rice, monosaccharides transport by OsMST1–8 has been demonstrated [46,47,85,86,87,88]. Among them, OsMST5, OsMST7, and OsMST8 have been shown to be involved in rice anther development [11,49,89].

## 5. Sugar Balance and Signaling Transduction

Plants photosynthesize carbohydrates by converting carbon dioxide into glucose during daylight, with sugar accumulation restricted by CO_2_ and light density [89,90]. Carbohydrates accumulate in leaves linearly through the day, peaking at dusk, but plants require energy throughout the nighttime hours as well. The plant circadian cycle regulates photosynthate partitioning to maintain energy balances in all plant tissues throughout the day and night [91]. In *Arabidopsis*, ~50% of the photosynthate accumulates as starch in the leaves during the day for degrading, to supply the sugar demands of non-photosynthetic tissues at night [92]. The rate of starch degradation at night is approximately linear, and ~95% of starch is utilized at the end of the night [93].

Further studies found that the circadian clock regulates the rates of starch degradation to coordinate the sugar supply and growth in *Arabidopsis* [91]. For example, three circadian-controlled transcription factors, PHYTOCHROME-INTERACTING FACTOR 3, 4, and 5 (PIF3, PIF4, and PIF5), precisely regulate the hypocotyl growth at the end of the night, which is an apparent energy-consuming process [94].The clock sets the pace of degradation to govern the starch exhausted at dawn, and this mechanism adjusts to photoperiod changes: when dark periods are extended from 12 h to 16 h, starch degradation occurs more slowly to reach the same minimum sugar levels at the end of the night [95,96]. A circadian-controlled gene, *SIGMA FACTOR5* (*SIG5*), encodes a transcription factor that controls the expression of several chloroplast genes, revealing the influence of core clock on the photosynthesis [97].

In addition to their fundamental functions in carbon and energy metabolism and polymer biosynthesis, sugars have feedback effects on the circadian clock [98]. In *Arabidopsis*, sugars gradually accumulate after sunrise at dawn, then repress the expression of PSEUDO-RESPONSE REGULATOR 7 (AtPRR7) and relieve repression on CIRCADIAN CLOCK-ASSOCIATED 1 (CCA1), modulating the phase of the circadian clock [98].

Sugars also act as signal transducers in a range of biological processes that are also modulated by hormones, such as seed germination, growth and development, and flowering [99,100]. Glucose has been shown to regulate genes involved in abscisic acid (ABA) hormone biosynthesis and signaling, which is antagonistic with the phytohormone ethylene [101]. An excess of sucrose can rescue a late-flowering phenotype as well as leaf morphogenesis and flowering in the dark. Sucrose, rather than auxin, levels play a vital role in apical dominance by suppressing the key regulator for bud dormancy, BRC1 [102]. Sugar accumulation also influences the juvenile–adult transition by suppressing the negative regulator miR156 and relieving repression on the *SQUAMOSA PROMOTER BINDING PROTEIN-LIKE* (*SPL*) genes, the promoting factor of miR172 [103,104].

## 6. Sugar Regulatory Network

Phloem loading is a complex process, achieved by a variety of pathways in different plants under different conditions; while the precise mechanisms in rice are not yet clear, a better understanding of this process may lead to improvements in grain production. Electron microscopy on rice leaf has revealed that xylem parenchyma cells are well-connected by plasmodesmata, suggesting the use of the symplastic pathway in rice phloem transport (Figure 1A) [105]. However, a later study suggests use of the apoplastic pathway (Figure 1B), as the expression of *AtSUC2* under control of the *Arabidopsis* Phloem Protein 2 promoter (*AtpPP2*) in rice enhances phloem loading and boosts grain yield by 16% compared with the wild-type [106]. *OsSUT1* is highly expressed in the phloem companion cells of source leaves, and the *ossut1* mutant shows defects in growth and grain filling [43]. Blocking sugar transmembrane loading in rice by overexpressing a yeast invertase limits normal growth and grain filling, proving that the apoplastic pathway plays an essential role in rice phloem loading [43]. In summary, the symplastic and apoplastic pathways both appear to play a role in rice phloem loading, and the relative importance of the two pathways and their specific mechanisms of action, specifically under different environmental conditions, would benefit from further study.

**Figure 3:**
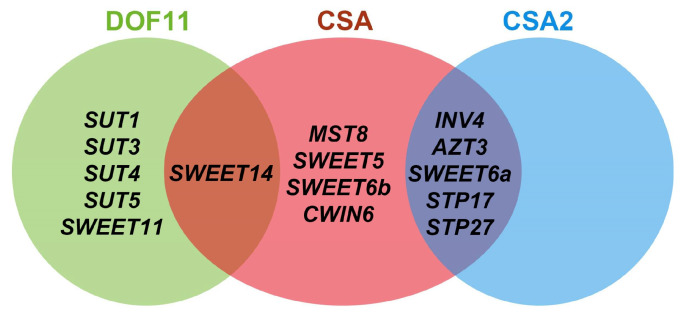
Sugar transport genes regulated by DOF11, CSA, and CSA2 in rice.

Several regulators of sugar transporters involved in the apoplastic pathway in rice have been identified, and suggest a complex network of regulation that responds to environmental conditions. Rice DNA BINDING WITH ONE FINGER 11 (OsDOF11) modulates sugar transport by regulating the expression of several sugar transporter genes, including *OsSUT1*, *OsSUT3*, *OsSUT4*, *OsSUT5*, *OsSWEET11* and *OsSWEET14*, and its mutant has defects in plant height and panicle development (Figure 3) [107]. Carbon Starved Anther (CSA) is reported to be a R2R3 MYB transcription factor regulating sugar transport during anther development, and also regulates *OsSWEET14* expression (Figure 3) [11,44]. The *csa* mutant reveals a photoperiod-sensitive genic male sterile phenotype due to the disruption of sugar supply to the anther under different photoperiods: male-sterile under short-day conditions but only semi-sterile under long-day conditions [11,12]. Interestingly, CSA2, another MYB transcription factor, reveals the reverse phenotypes, being fertile under short-day and semi-sterile under long-day conditions [13]. A further study suggests that CSA2 shares common downstream genes involved in sugar metabolism with CSA including *OsSWEET6a*, *OsINV4*, *OsAZT3*, *OsSPT17*, and *OsSPT27* (Figure 3) [13,44].

In plants, fluctuations in daylength are received in the leaves, and then a downstream signal, for instance, florigen, can be activated and move to other organs through vascular bundles [108]. Rice is a facultative short-day flowering plant, which can also flower under long-day conditions with a delayed flowering time [109,110]. *CSA* has a higher expression level in rice anthers under short-day conditions than long-day conditions, while the expression of *CSA2* reveals the opposite variations under different day-length conditions [12,13]. Transcriptome data reveal that core components of the circadian clock, *CCA1* and *PRR95*, play vital roles in sensing the photoperiod signals, and are then transduced to anthers [13,44]. CSA and CSA2 are likely to be regulated by these photoperiod signals, then control the source-to-sink transport via downstream sugar transporters to influence anther development (Figure 3) [13,44]. These results suggest the importance of these transcription factors in sensing environmental signals and regulating normal growth, especially anther development.

## 7. Conclusions

Hybrid rice has made great contributions to the increase in rice yield, meeting the food demands of expanding populations [111]. Understanding the molecular mechanisms of rice male reproductive development is crucial for hybrid rice breeding [4,5]. In this paper, we reviewed the source-to-sink transport processes of photosynthetic accumulation and the roles that sugar transporters play in the energy supply of the anthers during the male reproductive stages. While the overview of the regulatory network is limited, it provides new sights into how environmental signals influence sugar translocation via a series of transcription factors and proteins. In the future, new tools, including single-cell sequencing and hormone reporters, will provide new opportunities to elucidate the linkage of environmental clues, sugar transport and signaling, and male reproductive development [112,113]. The development of gene editing technology and crop breeding technology, such as CRISPR/Cas9 and Haploid-Inducer Mediated Genome Editing, will improve the understanding of the mechanisms and accelerate improvements in crop traits [114,115]. Research on the regulatory processes of sugar transport and their functions in male fertility have important implications for developmental biology and agricultural production.

## Figures and Tables

**Table 1:** Proteins involved in sugar metabolism in rice.

Gene Family	Number of Genes	Reported Genes/Reference
SUT	5	*SUT1* [38,39]; *SUT2* [40]; *SUT3* [41]; *SUT4, SUT5* [35]
SWEET	21	*SWEET5* [42]; *SWEET6a* [43,44]; *SWEET6b* [44]; *SWEET11* [15]; *SWEET14* [16]; *SWEET15* [45]
MST	64	
AZT subfamily	6	*AZT3* [13]
ERD subfamily	6	
pGlcT subfamily	4	
Xylose subfamily	2	
STP subfamily	15	*MST1*, *MST2*, *MST3* [46]; *MST5* [47]; *MST6* [48]; *MST8* [11]; *SPT17*, *SPT27* [43,44]
PLT subfamily	28	
INT subfamily	3	
Invertases	18	
VIN	2	*VIN2* [49]
CIN	8	*CIN8*/*Cyt-INV1* [50]
CWIN	8	*GIF1* [51]; *INV4* [10]; *CWIN6* [44]

The shaded areas represent subfamilies of these proteins.

## Data Availability

Not applicable.
